# Detection of Platelet-Activating Antibodies Associated with Heparin-Induced Thrombocytopenia

**DOI:** 10.3390/jcm9041226

**Published:** 2020-04-24

**Authors:** Brigitte Tardy, Thomas Lecompte, François Mullier, Caroline Vayne, Claire Pouplard

**Affiliations:** 1 Inserm U1059 Sainbiose, University of Lyon St Etienne, CIC 1408, FCRIN-INNOVTE, Hémostase Clinique CHU, 42055 Saint Etienne, France; 2 Department of Medicine, Geneva University Hospitals, and Geneva Platelet Group (GpG), Faculty of Medicine, University of Geneva, CH-1211 Genève 14; 3 CHU UCL Namur, Namur Thrombosis and Hemostasis center (NTHC), Hematology Laboratory, Université Catholique de Louvain, 5530 Yvoir, Belgium; 4 Department of Hemostasis, University Hospital of Tours, 37044 Tours, France; 5 University of Tours, EA 7501 GICC, 37000 Tours, France

**Keywords:** heparin-induced thrombocytopenia, diagnosis, functional assays

## Abstract

Heparin-induced thrombocytopenia (HIT) is a prothrombotic immune drug reaction caused by platelet-activating antibodies that in most instances recognize platelet factor 4 (PF4)/polyanion complexes. Platelet activation assays (i.e., functional assays) are more specific than immunoassays, since they are able to discern clinically relevant heparin-induced antibodies. All functional assays used for HIT diagnosis share the same principle, as they assess the ability of serum/plasma from suspected HIT patients to activate fresh platelets from healthy donors in the presence of several concentrations of heparin. Depending on the assay, donors’ platelets are stimulated either in whole blood (WB), platelet-rich plasma (PRP), or in a buffer medium (washed platelets, WP). In addition, the activation endpoint studied varies from one assay to another: platelet aggregation, membrane expression of markers of platelet activation, release of platelet granules. Tests with WP are more sensitive and serotonin release assay (SRA) is considered to be the current gold standard, but functional assays suffer from certain limitations regarding their sensitivity, specificity, complexity, and/or accessibility. However, the strict adherence to adequate preanalytical conditions, the use of selected platelet donors and the inclusion of positive and negative controls in each run are key points that ensure their performances.

## 1. Introduction

Heparin-induced thrombocytopenia (HIT) is a clinicopathological syndrome primarily caused by antibodies (Abs) of the immunoglobulin G (IgG) isotype that recognize platelet factor 4 (PF4) complexed with and modified by polyanions such as heparin (H). A rapid onset of the anti-PF4/H immune response and the simultaneous appearance of Abs of different classes (IgM and IgA) suggest short-term activation of B cells that have previously undergone Ig-class switching [1]. Pathogenic anti-PF4/H IgG Abs can induce strong platelet activation associated with an explosive generation of thrombin, which can lead to venous and/or arterial thrombosis. Monocytes, neutrophils, and endothelial cells are also activated by these antibodies; thus, HIT is characterized by multicellular activation [2,3,4,5]. Clinical diagnosis of HIT (including careful analysis of platelet count monitoring) is often complex, because patients are usually exposed to several causes of thrombocytopenia, especially when hospitalized [6]. Considering the major risk of thrombotic complications during HIT, a rapid and accurate laboratory diagnosis of HIT is essential in order to initiate or maintain a non-heparin anticoagulant therapy [7,8].

Laboratory tests used for HIT diagnosis must be integrated into a clinicopathological approach and are divided in two categories: immunoassays (IA), which detect antibodies directed against PF4 using labelled animal antibodies directed against human immunoglobulin, and functional assays, which measure heparin-dependent platelet activation/aggregation induced by HIT Abs [9]. Immunoassays and functional assays are in most cases used in combination and often sequentially as they suffer from different limitations. Indeed, immunoassays exhibit a very good negative predictive value and sensitivity, but have lower specificity and positive predictive value, since they detect both pathogenic and non-pathogenic HIT antibodies [10]. In contrast, functional assays are more specific, since they evaluate the heparin-dependent ability of HIT antibodies from patients to activate platelets from healthy donors, whether tested with platelet-rich plasma (PRP), isolated platelets, or whole blood. Moreover, functional assays are able to detect rare cases of HIT antibodies directed against other targets than PF4/H (like interleukin-8 (IL8) or neutrophil-activating peptide-2 (NAP-2)), which are undetectable by PF4-specific immunoassays [11,12]. However, a major issue about functional assays is their actual sensitivity. This review will focus on the most studied functional assays.

## 2. From Pathogenesis of HIT to Functional Tests for the Detection of Clinically Relevant Antibodies

HIT is primarily caused by the synthesis of IgG antibodies directed against modified platelet factor 4 (PF4), a positively charged protein released from α granules upon platelet activation, and which undergoes conformational changes when bound to electronegative macromolecules, such as heparin (H). Antigenic complexes of PF4/H are formed over a narrow molar range of PF4 and heparin (approximately 27 IU of heparin per mg of PF4), and an optimal ratio of the two components allows formation of ultra-large complexes (ULCs) (>670 KDa), which are particularly apt to be recognized by HIT antibodies [13]. In the presence of low concentrations of heparin (0.1 to 1.0 IU/mL), encountered during most types of heparin administration, ULCs are usually formed on platelet surfaces after binding to glycosaminoglycans (GAGs), such as chondroitin sulfate, and promote platelet activation induced by anti-PF4/H Abs, which bind to the FcγRIIa receptor [14]. In contrast, an excess of heparin induces the dissociation of ULCs, and HIT antibodies are no longer able to activate platelets [13]. The amount of PF4 on platelet surface is also critical, and data suggest that high levels of surface PF4 (>50 μg/mL) may be required for binding of HIT antibodies to platelets when patients are exposed to heparin [14]. This notion may partly explain why patients in post-surgery, especially after cardiopulmonary bypass (CPB), are particularly at risk of HIT, considering high levels of platelet activation and PF4 release in this context [15,16,17]. Furthermore, endogenous heparin could also induce conformational changes of PF4, which can be recognized by the HIT antibodies antibodies [14]. Thus, the release of large amounts of polyanions, such as deoxyribonucleic acid (DNA) or GAGs, may induce an immune response associated with the development of antibodies to modified PF4 even in subjects who have never been treated with heparin.

In whole blood, one of the main cellular targets of HIT antibodies is platelet; they express 80% of all FcγRIIa receptors [18]. Binding of the Fc fragment of HIT IgG antibodies to these FcγRIIa receptors triggers platelet activation, with the release not only of α granules inducing P-selectin expression on platelet surface, but also of dense granules, with serotonin, calcium, adenosine triphosphate (ATP) and adenosine diphosphate (ADP) release in the cellular microenvironment. This mechanism of activation finally leads to platelet aggregation. Importantly, gene polymorphisms affecting FcγRIIa (FcγRIIa H131R), or proteins involved in FcγRIIa-signaling, contribute to the variable response of platelets to HIT antibodies [19,20,21]. Indeed, these antibodies more efficiently activate platelets from subjects homozygous for the FcγRIIa 131R allele, but only when platelets are challenged in plasma or whole blood, due to the presence of normal IgG which compete with HIT antibodies for binding to FcγRIIa. This effect mainly depends on normal IgG2, which binds to the FcγRIIa 131H isoform more efficiently than to the 131R isoform. Importantly, platelet washing, which removes plasma environment, abolishes the impact of H131R polymorphism on platelet activation induced by HIT antibodies [20]. It should be emphasized that some HIT IgG subclasses are more common than others (IgG1 > IgG3 > IgG2 > IgG4) [15] and that the potential role of IgA and IgM antibodies has been occasionally mentioned but remains disputed [22].

In very few patients (<1%), other chemokines that share structural homology with PF4, such as IL-8 or NAP-2, could be involved [11,12]. However, unlike anti-PF4/H Abs, those directed against IL-8 and NAP-2 bind to their target in the presence or absence of heparin, and although addition of heparin enhances platelet activation, a high concentration of heparin does not systematically inhibit the binding.

**Figure 1:**
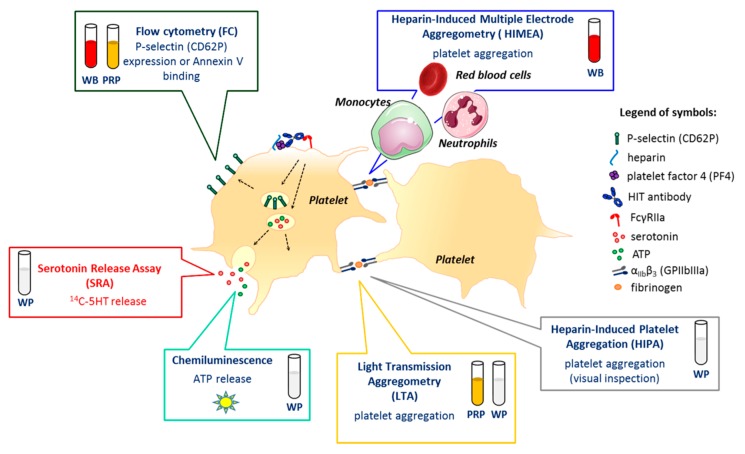
Platelet responses induced by heparin-induced thrombocytopenia (HIT) antibodies and functional assay targets. Depending on the assay, donors’ platelets are stimulated either in whole blood (WB), platelet-rich plasma (PRP), or after washings (washed platelets, WP). Today, serotonin release assay (SRA), which measures the release of serotonin from dense granules when platelets are activated, and heparin-induced platelet aggregation (HIPA), which is based on a visual inspection of platelet aggregates, are considered the “gold standard” to confirm HIT. The oldest but probably the most used assay is light transmission aggregometry (LTA), classically performed with PRP, but which can also be carried out with WP. Platelet aggregation can also be assessed in whole blood using heparin-induced multiple electrode aggregometry (HIMEA), which considers the possible contribution of monocytes, neutrophils, and red blood cells (RBC) to platelet activation induced by HIT antibodies. Dense granules secretion may also be evaluated by measuring the ATP released using chemiluminescence. Several assays based on flow cytometry (FC) have also been proposed, most of them assessing P-selectin (CD62P) expression or Annexin V binding to phosphatidylserine on the surface of activated platelets.

These elements of pathogenesis are crucial for understanding the design of HIT functional assays, as well as considering their limitations (Table 1; Figure 1).

## 3. Shared Aspects of Functional Assays

### 3.1. Test Platelet Preparation

All functional assays require fresh platelets from healthy donors, either in plasma environment (PRP), in buffered solution after washing steps (WP), or without any preparation when tested in whole blood (WB).

Platelet-rich plasma (PRP) must be prepared according to the International Society on Thrombosis and Haemostasis Scientific and Standardization Committee ISTH SSC recommendations provided for platelet testing [23] and the recent review published by M.C. Alessi et al. [24]. Blood samples should be drawn into a citrate anticoagulant (vol/vol 1/9) at 0.105 to 0.109 M final concentration and allowed to rest at room temperature for 15 min before the centrifugation step at low speed (i.e., 200 x *g* for 10 min at room temperature without brakes) [23]. The platelet-enriched supernatant is then harvested. After collection, PRP must be maintained for 15 min at room temperature before testing and the time elapsed between the blood drawing and the analysis should not be more than 4 h [23,24].

Washed platelets (WPs) are prepared from the blood collected in acid-citrate-dextrose (ACD) supplemented with either prostaglandin E_1_ (PGE_1_), apyrase, hirudin, or a mixture of them in order to prevent platelet activation and aggregation during the washing procedure [25,26]. The use of apyrase is recommended by many authors when washing platelets, since this enzyme degrades ADP and ATP, thereby preventing platelet desensitization to ADP, an important potentiator of HIT antibody-induced platelet activation [26,27]. Another approach to prevent the accumulation of ADP in platelet environment is the use of PGE_1_. Indeed, PGE_1_ increases adenylyl cyclase activity and therefore cyclic adenosine monophosphate (AMP) intra-platelet concentrations, inhibits calcium mobilization and platelet aggregation mediated by purinergic receptors [28,29].

Briefly, the PRP obtained as described above undergoes a fast centrifugation step (1100 g, 15 min) in order to drop platelets to the bottom of the tube and discard the plasma supernatant. Platelets are then washed by resuspension in a modified calcium-free and magnesium-free (in order to prevent platelet aggregation) Tyrode’s solution supplemented with bovine serum albumin and PGE_1_, apyrase, or hirudin. After centrifugation, platelets are finally suspended in a Tyrode’s solution containing a physiological concentration of calcium [30].

### 3.2. Platelet Donor Selection

It is well established that the source of platelets and their preparation conditions have a considerable influence on functional assay results [20,31], and to avoid any misinterpretation of them, several pre-analytical variables need to be controlled [23]. Platelets should be collected from healthy donors after a short period of rest, at least 30 min without smoking and 2 h after caffeine ingestion. Donors should be free from any medications that could affect platelet function, such as non-steroidal anti-inflammatory drugs (for 3 days at least), aspirin (for 10 days), selective serotonin reuptake inhibitors, or several herbal treatments (quinine, cumin, dong quai, fenugreek, garlic, onion, ginger, ginseng) [32]. As it is not known how long it takes to restore platelet function after these molecules have been discontinued, it may be preferable to exclude platelet donors who use these substances. Moreover, some factors intrinsic to platelets may also affect their reactivity, and explain the inter-donor variability of response. Importantly, blood group ABO status seems inconsequential [33]. In contrast, FcγRIIa H131R polymorphism has a major impact on platelet reactivity to HIT antibodies in a plasma environment, with H/H platelets being less sensitive [20]. To overcome this issue, selection of platelet donors may be useful. Some authors proposed the use of a murine anti-CD9 monoclonal antibody (ALB6) that cross-links FcγRIIa receptors [34], but this approach has limitations since ALB6 is a murine IgG_1_ antibody, therefore, features very low affinity for the 131H isoform of human FcγRIIa [35]. In practice, some authors proposed to dilute a well-characterized strong HIT serum (typical activation profile, short lag time, activation of most of platelet donors) to provide “weak positive” controls to validate platelets reactivity [10,26] due to the lack of guidelines for the selection of platelet donors. The monoclonal anti-PF4/H IgG antibody 5B9, which has a human Fc portion, is potentially a good tool to select platelet donors [36]. Importantly, the intrinsic reactivity of platelets of normal subjects is stable over time and it is thus possible to select platelets from the donors already known to be sensitive to HIT antibodies (the so called “good responders”) [26]. Using this approach, platelets of one or two donors only are required for each run [37]. On the other hand, when platelets are used without donor selection, another way to overcome platelet response variability is to prepare platelets from a higher number of donors. There is no general consensus on the number of donors to be tested in each run; the suggested numbers of random donors vary from two to five [27,33,38].

### 3.3. Preparation of Samples from Patients with Suspected HIT

The use of citrated platelet-poor plasma (PPP) or serum samples does not seem to matter much for HIT testing. As with all the tests based on detection of IgG Abs, samples can be stored frozen between −20 °C and −80 °C before testing. Noticeably, immunoglobulins are stable in undiluted samples stored at −80 °C for at least 3 years [39].

Plasma sampling with CTAD (a mixture of citrate, theophylline, adenosine, and dipyridamole) must be avoided, as this mixture inhibits platelet activation [40,41]. Plasma or serum samples should be prepared from whole blood by centrifugation at 1500 g for 15 min.

Serum (and to a lesser extent, plasma) may contain residual thrombin that activates platelets and therefore heat inactivation (56 °C for 30 min) followed by high-speed centrifugation (8000 *g* for 15 min) is highly recommended [9,42]. It is important to keep in mind that serum contains more PF4 than plasma due to its release from α granules upon platelet activation by endogenous thrombin, which may facilitate initial formation of antigenic complexes [43,44].

Importantly, HIT antibodies disappear on average within 3 months after the onset of HIT when detected using immuno-enzyme assays, but functional tests tend to become negative earlier [45]. The median time to a negative test is 50 days for functional assays and 85 days for immunoassays. It is therefore recommended that sample collection be performed in the acute phase of HIT (day of suspicion) and before starting anticoagulation with danaparoid, as this drug may render functional tests negative [46,47]. The recent works suggest that treatment with ticagrelor may also cause false negative results [48]. On the other hand, the presence of direct thrombin inhibitors does not seem to influence results [47]. 

The presence of heparin in a patient’s sample may interfere with functional assays by modifying the final concentration of heparin in the reaction mixture [49]. It is therefore recommended to collect blood at least 4 h after heparin infusion is stopped or just before the next subcutaneous injection [34]. The use of heparinase has been proposed when urgent HIT testing is necessary for patients receiving heparin [50], but such a procedure is not fully validated. The measurement of anti-Xa activity in the plasma sample may also ensure the absence of heparin.

### 3.4. Heparin Concentrations

The concentration of unfractionated heparin (UFH) that enables maximum platelet activation when HIT antibodies are present ranges from 0.1 to 1 IU/mL, depending on the functional assay [51]. Low heparin concentrations (0.1–0.3 IU/mL) are optimal for washed platelets-based assays, whereas higher concentrations (0.5–1.0 IU/mL) may be needed for the assays performed with PRP and whole blood [33,52,53]. Moreover, the optimal concentration of heparin may vary from one donor to another, possibly due to variations of PF4 density on platelet surface [14]. Consequently, in order to increase sensitivity of the assay, it is recommended testing two different concentrations of heparin within the range observed in most clinical settings [26,27,33]. Some teams directly used the heparin batch administered to patients for increasing assay sensitivity, but this approach has not been validated. It has also been proposed to evaluate in vitro cross-reactivity of HIT antibodies with danaparoid [54]. However, it is important to note that in vitro results do not correlate well with in vivo cross-reactivity [55].

Importantly, as a feature of HIT antibodies is their incapacity to activate platelets in the presence of high concentrations of heparin, one condition using a high concentration of heparin is mandatory when performing HIT functional assays to evidence a “heparin-dependent” activation pattern. This condition also increases specificity of assays, as anti-platelet antibodies with other specificities, such as anti-human leukocyte antigen (HLA), may also induce platelet activation, but independently of the presence of heparin, and are therefore identified by persistence of “non-specific” activation at high heparin concentrations [56]. A concentration of 10 to 100 IU/mL is generally used in most functional tests with the exception of Heparin-Induced Multiple Aggregometry (HIMEA), for which a concentration of 200 IU/mL is suggested [26,33,34].

### 3.5. Platelets, Patient Samples, and Heparin: Volumes to Be Used

Protocols of functional assays are different from one another. Therefore, the table below lists the most frequently used volumes of major components (platelets, patient’s sample, and heparin) in each test (Table 2).

## 4. Optical Aggregometry

Platelet aggregation tests (PAT) using light transmission (optical) aggregometry (LTA) have long been used to detect platelet-activating HIT Abs [57]. This test is widely used in specialized laboratories that also resort to this approach to diagnose platelet function disorders, but its reported performance for detecting HIT antibodies varies from one study to another (Table 3) [33,58,59,60]. However, when pre-analytical and analytical recommendations (i.e., donor selection, positive control, high heparin concentration) are rigorously applied, the performance of this assay may approach that of the Serotonin Release Assay (SRA), although remaining slightly lower. Indeed, Chong et al. report a sensitivity of the SRA ranging from 65% to 94% depending on the platelet donor compared to a sensitivity range of 29% to 82% for PAT using the same platelets donors [33]. However, a recent study observed a clearly inferior sensitivity of the PAT done with PRP compared to the washed platelet Heparin-Induced Platelet Aggregation (HIPA) when the rules for HIPA positivity (2 positive out of 4 platelet donors) are applied to PAT [61].

In practice, PRP from a healthy subject is incubated with patient plasma or serum at 37 °C for 15 to 30 min under constant stirring (1100 rpm) without and with various concentrations of heparin (UFH– 0.5, 1.0, and 100 IU/mL, final concentrations). Some teams use equal volumes of normal PRP and the test sample, and others a ratio of 2 volumes of PRP for 1 volume of the test sample. Furthermore, some HIT samples may display the best reactivity with 0.5 IU/mL heparin, while others do with 1.0 IU/mL heparin. It is therefore recommended that two pharmacologic concentrations of heparin (0.5 and 1.0 IU/mL) be systematically tested to increase sensitivity of the assay [33]. 

**Figure 2:**
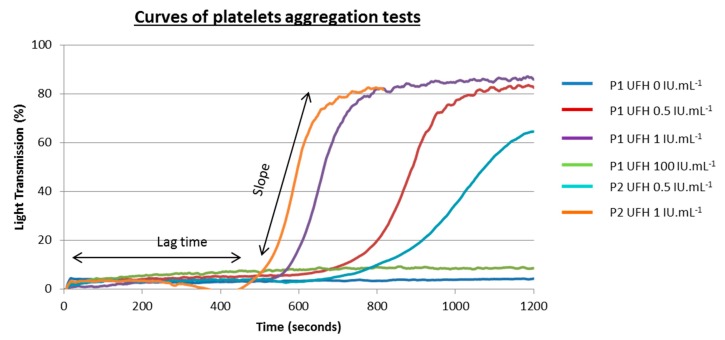
Typical tracings obtained with light transmission aggregometry in the absence and in the presence of low (0.5 and 1 IU/mL) and high (10 IU/mL) unfractionated heparin UFH concentrations. Aggregation curves for two patients with HIT (P1 and P2) are presented here. Different lag times related to heparin concentrations and patient samples are presented. P1 antibodies appear to be more reactive than P2 antibodies.

Platelet aggregation is reflected by an increase in light transmission through a platelet suspension as macro-aggregates form. A positive PAT curve is defined by a platelet aggregation of more than 25% obtained with therapeutic concentrations of heparin [33]. But, in practice, platelet aggregation of more than 40–50% is expected with sigmoidal tracing, which is a very suggestive aspect (Figure 2).

Because platelet reactivity may vary from one donor to another, platelet sensitivity to HIT antibodies must be checked in each run using a weak positive HIT plasma control (a 1/2 to 1/3 diluted strong positive HIT sample) [31].

It has been proposed that before being considered negative, PAT should be performed with at least two different donors (who tested positive with the HIT positive control) [38]. In case of high suspicion of HIT and negative PAT with several donors, consideration may be given to performing PAT with patient’s platelets after heparin (and/or danaparoid) withdrawal, and once platelet count has recovered. In these experimental conditions, specificity of the reaction has to be checked in presence of a high heparin concentration, or testers ought to resort to a more sensitive test using WP. 

Of note, though, it has been shown that a high heparin concentration (100 IU/mL) may also inhibit platelet aggregation induced by other activators, such as ADP, collagen, and epinephrine [38], thus deserving some caution in the interpretation of negativity.

## 5. Flow Cytometric Assays

Flow cytometric assays (FCA) are based on detection of activation markers on platelet surface using fluorescence-labeled ligands that emit a fluorescence signal proportional to the binding to their targets. Among these markers, the most commonly used is P-selectin (CD62P), a component initially present in the α granule membrane, and phosphatidylserine (PS), a negatively charged phospholipid exposed by platelets upon activation and detected with annexin V binding [63,75].

**Figure 3:**
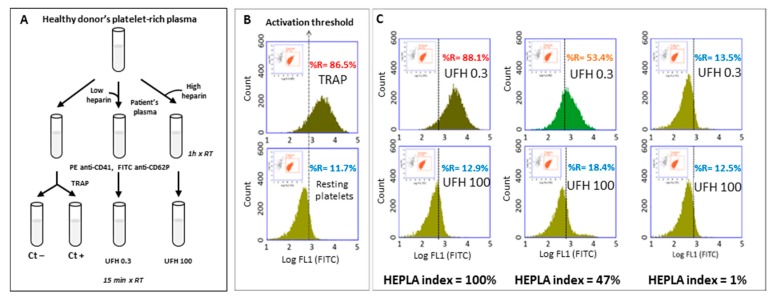
Detection of heparin-dependent platelet-activating antibodies with FCA. (**A**) Schematic procedure of the FCA. (**B**) The first gate delineates the platelets ((log SSC + log FL2-PE (CD41+ events)) and the second gate is applied to analyze the activated platelets (log FL1-FITC). Determination of the activation threshold: upper panel, TRAP-activated platelets (Ctl+); lower panel, resting platelets (Ctl−), the cursor indicating the activation threshold is placed at the intersection of the FL1 histograms of the positive control (Ctl+) and the negative control (Ctl−). “%R” represents the percentage of CD-62P positive events as an index of platelet activation. This set-up allows determining percentage of the activated platelets (to the right of the activation threshold) under different conditions. (**C**) Incubations with patients’ plasma and one platelet donor: typical results with low (0.3 IU/mL heparin) and high (100 IU/mL heparin) concentrations of heparin (top and bottom rows, respectively). Left panels: platelets activated with a highly HIT-positive plasma (optical density, OD = 2.5); middle panels: platelets activated with a weakly HIT-positive plasma (OD = 1.3); right panels: platelets incubated with a non-HIT plasma. The HEPLA index is calculated as follows: HEPLA index = (% H 0.3 − % H 100)/(% TRAP Ctl+ − % PBS Ctl−) × 100. FCA: flow cytometric assay, SSC: sideways scatter, PE: phycoerythrin, FITC: fluorescein isothiocyanate, TRAP: thrombin receptor agonist peptide, UFH: unfractionated heparin.

Flow cytometric assays are carried out according to a methodology first described by Tomer and performed with PRP, and possibly with whole blood [63,75,76]. Patient and control samples are incubated with donor PRP and either with phosphate buffer saline (PBS), low (0.1 and 0.3 IU/mL^−1^), or high (100 IU/mL^−1^) concentrations of UFH. Each reaction tube is then further incubated at room temperature with a mixture of phycoerythrin (PE)-conjugated anti-CD41a (to identify platelet population) and fluorescein isothiocyanate (FITC)-conjugated anti-CD62P monoclonal antibodies (mAbs), or FITC-conjugated annexin V identifies activated platelets [67,68,69]. Platelet activation and labeling is then stopped by addition of a PBS buffer, and samples are immediately analyzed by flow cytometry. In each run, donor platelet reactivity can be assessed with the addition of thrombin receptor agonist peptide (TRAP) or calcium ionophore (positive control (Ctl+)). The level of spontaneous platelet activation is also assessed on a mixture of donor PRP + donor, PPP + PBS as a negative control (PBS Ctl−). Platelet sensitivity to HIT antibodies is assessed using HIT-positive control plasma samples (HIT Ctl+) obtained from patients with confirmed HIT (Figure 3). 

Nowadays, two functional assays based on the FCA method are marketed: Emo-test HIT confirm^®^ (Emosis, Illkirch-Graffenstaden, France) and HIT Alert^®^ (IQ products, Groningen, Netherlands), which are based on P-selectin (CD62P) and PS expression on platelet surface, respectively. Platelet suspensions may be analyzed with a standard flow cytometer equipped with two light scatter detectors and four fluorescence detectors only, and the simpler the equipment, the easier are the settings. Fluorescence compensation has to be applied to correct emission spectrum overlap between FITC and PE using single color-labeled preparations. Logarithmic side scatter (SSC) versus Log FL2 (PE anti-CD41a) gating allows differentiation between the platelet population (CD41a^+^) and the cell debris (CD41a^-^). Activated platelets are identified by FITC anti-CD62P mAbs or annexin V. Regarding the Emo-test HIT confirm^®^ assay, platelets are analyzed according to the histogram defined by counts versus logarithmic FITC fluorescence (FL1). A total of 10,000 platelets (CD41^+^ events) are analyzed in each sample and the percentage of activated platelets is evaluated (FL1 histogram). For each run, a cursor indicating the activation threshold is placed at the intersection of the FL1 histograms of the negative control (Ctl−) and the positive control (Ctl+). This set-up allows determining percentage of the activated platelets (to the right of the activation threshold) under four different conditions: buffer (Ctl−), TRAP (Ctl+), patient PPP with 0.3 IU/mL heparin (H 0.3) and 100 IU/mL heparin (H 100). 

Recently, a standardization of the expression of platelet activation has been proposed [68] and can be used for interpreting Emo-test HIT confirm^®^ results. It is based on the calculation of the heparin platelet activation (HEPLA) index defined as follows: HEPLA index = (% H 0.3 − % H 100)/(% TRAP Ctl+ − % PBS Ctl−) × 100. 

If residual heparin is present in the patient’s sample, basal platelet activation may be observed before addition of heparin to the mixture. In case of HIT, no platelet activation should be observed in the presence of 100 IU/mL UFH, and this point is therefore more relevant than the one without heparin for HEPLA calculation, allowing a reliable conclusion even in the presence of residual heparin in the plasma. A plasma sample should be considered HIT-positive if the HEPLA index is higher than the cut-off value with at least one platelet donor out of the 2 to 5 tested depending on the clinical probability of HIT.

Several studies evaluated performance of the FCA, with reported sensitivity varying from 70 to 100%, and specificity—from 75 to 100% (Table 3) [63,64,65,70,75]. However, with whole blood, one study reported a much lower sensitivity of the FCA (38%) for detecting the HIT without thrombosis, but an excellent sensitivity (higher than 90%) for the HIT associated with thrombosis [76].

Importantly, the absence of the requirement to wash platelets when using the FCA makes this assay less demanding [77]. 

## 6. Heparin-Induced Platelet Activation (HIPA)

The HIPA test developed in 1991 is based on a visual assessment of platelet aggregation every 5 min in a microtiter plate without an aggregometer [27]. This assay is performed with washed platelets adjusted to 300–400 G/L in the Tyrode’s solution and kept for 45 min at 37 °C before testing. Platelets are then incubated with a heat-inactivated patient’s sample in the presence of a buffer (Ctl−), therapeutic (0.2 IU/mL reviparin) or supratherapeutic concentrations of heparin (100 IU/mL UFH) in a round-bottom microtiter plate. The authors who first described this test used reviparin, since this low molecular weight heparin (LMWH) has a narrow range in molecular weights, which would provide more consistent formation of PF4/heparin complexes, thus improving assay sensitivity. Magnetic stirrers are used to promote platelet-platelet collisions and thus favor aggregation if activated, and formation of aggregates is visually determined every 5 min. Wells are examined against an indirect light source: a change in appearance of the reaction mixture from turbidity to transparency indicates platelet aggregation. The test is considered positive if aggregation is observed within 30 min in the presence of therapeutic but not high concentrations of heparin with at least two of four unselected platelet donors, a number empirically chosen by the authors. As for other functional tests, positive and negative controls must be included in this assay. While the buffer can be a negative control, it has been proposed that positive control of HIPA be an HIT-positive serum diluted with normal serum until reaching a lag time of approximately 25 min for platelet aggregation [71].

HIPA is considered the “gold standard” by some [42], but this assay was compared to the SRA in only few studies carried out by the same team [27,78] (Table 3). The international pilot external quality assessment (EQA) of HIT diagnosis, including functional assays (SRA and HIPA), recently found an agreement for 70% of HIT-positive samples only [78].

In a prospective study, Greinacher demonstrated that HIPA and PF4/H enzyme-linked immunosorbent assay (ELISA) are sensitive tools for HIT diagnosis [59]. Moreover, HIPA offers the advantage of a faster turnaround time (results reported within 24 h in Germany) compared to the “gold standard” assay, SRA, and does not necessitate the use of radioactivity. 

Importantly, the criteria for test positivity are challenged in patients from intensive care units (ICU) for whom false positive results have been described [79]. 

## 7. Whole Blood Aggregometry

Difficulties related to the numerous pre-analytical and analytical variables to be considered when using PRP or WP and having an impact on the performance of the LTA or HIPA led to the development of whole blood impedance aggregometry (WBIA) performed with a Multiplate^®^ analyzer (Roche, Rotkreuz, Switzerland). This instrument was first developed for monitoring antiplatelet therapies and measures changes in conductivity between electrodes (two sets) as activated and aggregated platelets stick to them. Several teams adapted this instrument to HIT diagnosis and developed a simple and rapid functional assay called the “heparin-induced multi-electrode aggregometry method” (HIMEA) [62,72,74,80] (Table 3). Of note, normal WB anticoagulated with hirudin is mixed with patient’s citrated plasma or serum.

**Figure 4:**
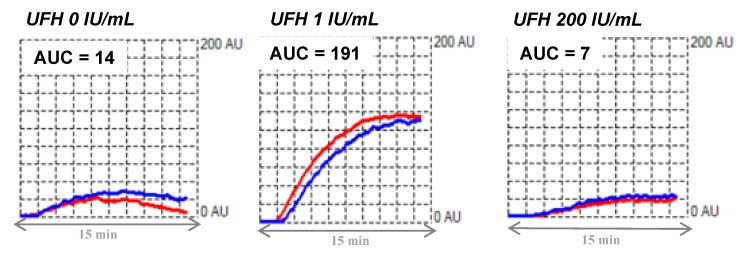
Typical tracing obtained by whole blood impedance aggregometry. Presence of HIT antibodies activating platelets: typical tracings obtained by whole blood impedance aggregometry (Multiplate^®^) in the absence and in the presence of low (1 IU/mL) and high (200 IU/mL) concentrations of UFH. The whole blood aggregation is recorded for 15 min. AUC: area under curve.

In 2016, the platelet immunology subcommittee of the ISTH formulated recommendations for standardization of this assay for HIT diagnosis [34]. Fresh blood should be collected using hirudin as an anticoagulant (25 μg/mL final concentration) rather than tubes filled with citrate, as this improves sensitivity of this assay, with shorter lag time, increased velocity and area under the curve (AUC) [53]. Importantly, blood should be left at rest for ≥30 min before testing. Then, WB is incubated in the analyzer under stirring at 37 °C for 1 min with the patient’s plasma or serum and a saline solution. This step is very important to reduce background noise. Saline buffer, therapeutic (1 IU/mL) and supratherapeutic (200 IU/mL) concentrations of heparin are further added into the reaction mixture and impedance changes are recorded for 15 min. The high concentration of heparin recommended here (200 IU/mL) is higher than that used in other functional assays, because 100 IU/mL of heparin may not be sufficient to completely inhibit platelet activation by HIT antibodies in the HIMEA [53]. On the other hand, it may be appropriate to perform additional testing using 0.5 IU/mL heparin in case of borderline results with 1 IU/mL, as this may improve results in samples with low-titer antibodies [53]. All experiments should be run within 3 h to obtain reliable results, which are expressed as the area under the curve (AUC) using arbitrary units. Results are considered positive if they fulfill all the following criteria (Figure 4): (1) a typical sigmoidal curve reflecting actual platelet aggregation; (2) AUC (1 IU/mL UFH) > 30 U; this cut-off has to be defined in each center (based on the measurements performed on 20 healthy controls and non-HIT patients treated with heparin); (3) AUC (200 IU/mL UFH) < 50% of the AUC obtained with 1 IU/mL heparin; (4) AUC < 30 U without heparin (threshold to be defined in each center). 

The fourth item is questionable, as patient samples may contain residual heparin or atypical HIT antibodies that may induce platelet activation without heparin [81]. 

The advantages of HIMEA include rapid turnaround time and ease of completion. Moreover, carried out with whole blood, HIMEA does not require sample handling, thus minimizing artefactual platelet activation. In this context, using a known reactive platelet donor, Morel-Kopp et al. reported an improved performance of HIMEA compared to LTA (PAT) for HIT diagnosis, with results comparable to those of SRA [53]. Similar results were recently published by Jing Jin et al., with sensitivity of 85% and specificity of 98% attributed to the HIMEA, both not statistically different from those of SRA [73]. Of note, in this study, the same high responder donors were used for both functional assays, which was ideal for method comparison.

### Potential Pitfalls in Interpretation of the HIMEA

It is important to keep in mind that absence of the characteristic lag phase, variable results between replicates, and strong platelet aggregation (AUC > 300) without heparin are more suggestive of heparin-independent aggregation than the presence of HIT antibodies [73].

## 8. Serotonin Release Assay

In order to increase sensitivity of HIT functional assays, it has been proposed to evaluate an early stage of platelet activation, i.e., granule release, rather than platelet aggregation, since antibodies may induce granule release without aggregation [82]. Therefore, in 1986, Sheridan et al. developed a functional assay called serotonin release assay (SRA) performed with washed platelets and measuring dense granule release [52]. Serotonin (or 5-hydroxytryptamine, 5HT) is a neurotransmitter stored in the dense granules of platelets and released upon activation. Briefly, WB from healthy subjects is collected on the citric acid–citrate–dextrose (ACD) anticoagulant with PGE_1_ and/or apyrase, and after centrifugation, PRP is collected and incubated with ^14^C-radiolabeled serotonin (0.1 μCi/mL of PRP) for 45 min at room temperature (approximatively 50% of ^14^C serotonin is incorporated in dense granules). Platelets are then washed (see above), resuspended in the Tyrode’s solution with calcium to the final platelet count of 300 G/L, and incubated with the patient’s sample for 1 h at room temperature in microtubes, without and with various concentrations of heparin (0.1, 0.5, 1, and 10 or 100 IU/mL). Platelet activation is then stopped with the PBS buffer supplemented with ethylenediaminetetraacetic acid (EDTA), followed by high-speed centrifugation of the samples and measurement of the supernatant radioactivity using a scintillation counter. In each experiment, total platelet radioactivity (max radioactivity) is defined by a condition in which platelets are lysed with 0.5% Triton-X100. In addition, the buffer was substituted for the patient sample in order to obtain the background radioactivity level (min radioactivity). The percentage release of serotonin is finally calculated using the following formula: (radioactivity of the sample) − (min radioactivity)/(max radioactivity) − (min radioactivity). The result is considered positive if the release is >20% measured with 0.1 to 1 IU/mL heparin, associated with inhibition of serotonin release (release < 20% or inhibition ≥ 50%) with 10 or 100 IU/mL heparin. HIT serum usually causes strong platelet activation (>80% serotonin release) at low heparin concentration, with a smaller proportion causing maximal release between 50 and 80%; even fewer SRA-positive sera will cause only weak serotonin release (20–50% release) [26]. There may be some variability from laboratory to laboratory regarding the platelet washing procedure, which explains at least in part why some request a 50% threshold for serotonin release and others only 20%. Of note, the SRA may also be performed in microtiter plates, which is particularly suitable for multiple testing [26]. A major drawback of the SRA is the requirement of radioisotopes that are nowadays progressively avoided for regulatory and safety reasons. For that reason, it has been proposed to assess serotonin release using high-performance liquid chromatography [83].

Today, the SRA is considered the “gold standard” for HIT diagnosis because of its excellent specificity (>99%) and good sensitivity (88–95%) [26,52,60,66] (Table 3). However, it is important to emphasize that there is no reference standard for evaluating performance of the SRA [7], and validation should be performed against the classification performed by experts according to the explicit diagnostic criteria in a blind manner. In addition, an extensive study was conducted on the variability of SRA results among platelet donors, testing 10 HIT-positive serum samples with platelets from 10 healthy donors. The authors of this study demonstrated that platelet donor variability and serum HIT antibody concentration significantly affected SRA results. Indeed, false negative results were observed when low levels of HIT antibodies were mixed with poorly reactive platelets [31].

Recently, sensitivity of the SRA has been brought into question by two independent groups [84,85]. Vayne et al. showed that sensitivity of the SRA was improved by addition of exogenous PF4 to the platelet buffer, thus highlighting that the PF4 amount on platelet surface is a critical factor for detecting HIT antibodies, particularly when using washed platelets [85]. For this reason, the authors recommend adding human PF4 (10 μg/mL) to WP when performing the SRA in order to detect low levels of pathogenic HIT antibodies. 

## 9. Other Functional Assays

There are still other possible functional assays in addition to those mentioned above.

Apart from serotonin, dense platelet granules also contain large amounts of adenosine triphosphate (ATP), which is released upon activation, and can be measured in PRP or with washed platelets using a lumi-aggregometer [86].

HIT is characterized by FcγRIIa-dependent platelet activation associated with proteolysis of this receptor for IgG, and thus it has been proposed that a FcγRIIa proteolytic fragment may serve as a surrogate marker for HIT [87]. Although this assay seems to be at least as specific as the SRA, it still requires preparing fresh washed platelets. 

It should be relevant to look at platelet procoagulant activities and emission of procoagulant extracellular vesicles [12,88]. Assessment of thrombin generation with PRP was reported to have an HIT diagnostic performance comparable to other tests performed with PRP [89]. Further studies along this path deserve to be done.

In a much less affordable way, another assay proposed to substitute human platelets by a transgenic B-cell line expressing FcγRIIa coupled to a luciferase reporter [90].

## 10. Shared Difficulties when Interpreting Functional Tests

### 10.1. Persistent Platelet Activation with Supratherapeutic Heparin Concentration

Some patient samples may induce platelet activation with both low (0.1 to 1 IU/mL) and high (10–100 IU/mL) heparin concentrations. When the assay is repeated using another heat-inactivated aliquot, interpretable results are often obtained, suggesting that the first results may have been impacted by artefacts such as aggregated IgG generated in vitro [26]. However, some samples definitely exhibit such a pattern, which could be due to circulating immune complexes, high titers of allo-antibodies to HLA class I antigens, or other platelet-activating factors [26]. Such results must be considered a “non-specific platelet activation”.

### 10.2. Interpretation of Platelet Activation by HIT Antibodies in the Absence of Heparin

When performing HIT functional assays, it is not uncommon to observe platelet activation with HIT samples even in the absence of added heparin (positive buffer pattern), but associated with complete inhibition of platelet activation with supratherapeutic concentrations of heparin. Two potential explanations can be proposed. First, there may be residual heparin in patients’ samples, capable of promoting platelet activation by HIT antibodies. Although digestion with heparinase I and/or isolation of HIT immunoglobulins have been proposed, such approaches may prove ineffective to overcome this artefact, indicating that positive buffer pattern is not always due to heparin contamination [91].

The second explanation for platelet activation induced by HIT antibodies without heparin has recently been proposed by Greinacher et al. who described a subset of “auto-immune” HIT antibodies able of inducing such an atypical platelet activation pattern [81]. In vitro, these antibodies have a surprising ability to bind two different PF4 tetramers with each of their two Fab arms, thus forcing them together, which results in critical conformational changes of PF4, similar to those observed with heparin, thus promoting binding of classical HIT antibodies to platelets [92]. Importantly, such reactivity predicts greater pathogenicity of HIT antibodies, including greater magnitude of thrombocytopenia, higher frequency of HIT-associated disseminated intravascular coagulopathy (DIC), and longer time to platelet count recovery [26,93].

## 11. Quality Controls

In order to make sure that platelet activation is specifically related to FcγRIIa engagement by HIT IgG, a monoclonal antibody, IV.3, which specifically inhibits this receptor, might be used. Therefore, platelets are pre-incubated with IV.3 that blocks access of HIT antibodies to this receptor. However, this inhibitory control is only specific to the FcγRIIa activation pathway, which can be activated independently of HIT. To date, no specific internal quality control is available for HIT functional assays, and it is therefore highly recommended to test reactivity of donors’ platelets to HIT antibodies with at least a weak positive HIT control [26]. Of note, HIT sera may be stored for a long time frozen at −70 °C with persistent reactivity, but iterative freeze/thaw cycles may induce formation of IgG aggregates that may induce non-specific platelet activation. It is therefore preferable to aliquot HIT-positive control samples for better preservation. Functional tests can only be validated if the HIT control is positive with all the tested donors. 

In the future, a HIT-mimicking monoclonal antibody may be used as the positive control. Indeed, Gruel’s team recently developed the first chimeric monoclonal HIT antibody with a human Fc fragment (5B9), which behaves in a similar way to human anti-PF4/heparin IgG antibodies [94]. An ISTH grant has just been obtained to evaluate whether this monoclonal antibody could be used as the positive control for HIT platelet activation tests and platelet donor selection. The five most common functional assays (SRA, PAT, HIMEA, HIPA and FCA) will be evaluated in an international multicenter study. 

The pilot external quality assessment study outlined how difficult it is to organize such a study with samples from patients. The negative sample consisted of the pooled serum from AB-donors, which probably contains aggregates of antibodies or immune complexes, thereby causing discrepancies. Furthermore, it is difficult to obtain large enough patient samples to run such a study with many participants [78].

## 12. Comparison of Washed Platelet and PRP Activation Assays

A direct comparison of PAT simultaneously performed with PRP and WP using the same platelet donors and the anti-PF4/H IgG antibody (5B9) found a higher sensitivity of washed platelets for detecting HIT antibodies [20]. The competition between HIT antibodies and normal plasma IgG may explain, at least in part, the low sensitivity of PAT performed with PRP compared to the assays performed with washed platelets, such as HIPA or SRA [61]. However, Pouplard et al. reported a similarly high sensitivity of the PAT performed compared to the SRA (91% vs. 88%) when good responder donors were selected [60]. It is therefore important to note that when a PAT is performed with platelet donors’ selection and control samples, performance of this assay may be significantly improved [33]. A recently published study evaluated performance of the four main functional assays (PAT with PRP, PAT with washed platelets, FCA, HIMEA) in comparison with the SRA using monoclonal anti-PF4/H IgG antibody 5B9. The authors concluded that the SRA likely remains the most sensitive and specific assay for detecting platelet-activating HIT antibodies, but HIMEA or FCA are potential alternatives, despite being less performant [36].

## 13. Discussion

In most patients, HIT cannot be definitively diagnosed on the basis of clinical criteria alone. Importantly, pathogenic heparin-dependent antibodies are detected in only 10–15% of patients with HIT suspicion, highlighting the importance of performing laboratory assays capable of demonstrating both presence and pathogenicity of heparin-dependent antibodies. Positivity of a functional assay may require only one positive platelet donor, but the number of platelet donors to be tested before excluding HIT is not well defined, and depends on the type of the functional assay. Although sensitivity of PRP-based assays is higher when multiple donors are tested, the most sensitive assays remain those performed with washed platelets; they can also be even more sensitive with the addition of exogenous PF4, even though clinical relevance of such Abs is still debated [95]. Whatever the test used, its specificity should be assessed by inhibition of platelet activation in the presence of high concentrations of heparin. Moreover, FcγRIIa-mediated platelet activation by HIT IgG antibodies can also be inhibited by the addition of monoclonal antibody IV.3 directed to FcγRIIa. It is important to keep in mind that functional testing can be performed with frozen samples collected prior to the replacement therapy, and international recommendations suggest that patients be treated without waiting for the results of functional tests [96,97]. Most specialized hemostasis laboratories should be able to perform HIT functional assays, the best being the one they are most familiar with, provided they meet all the controls described above. Conversely, a central experienced and efficient lab can serve a given area. 

Functional tests should always be integrated into a clinical laboratory approach and should be performed after immunoassays using the Bayesian approach [7,98,99]. In the vast majority of cases, they are performed only when an immunoassay is positive to evidence the ability of the detected anti-PF4/H Abs to activate platelets. The latest American Society of Hematology (ASH) guidelines, as well as those proposed by the French Working Group on Perioperative Haemostasis Groupe d’intérêt en hémostase périopératoire (GIHP) even go so far as to suggest that a functional test is not required for patients with a high clinical probability (4Ts score) and a very highly positive immunoassay (e.g., an optical density of 2.0 in ELISA) [96,97]. However, such an approach has only been validated using ELISA with PF4/polyvinyl sulfonate coated in wells, and may not be applicable with other ELISA, as suggested by the significant variations observed in OD values from one assay to another using the same HIT sample [100].

In some cases, patients with HIT may have a negative immunoassay, either due to a laboratory error or because the antigen involved is a chemokine different from PF4 (IL8 or NAP-2) or is improperly exposed in the solid phase [101]. 

## Figures and Tables

**Table 1:** Methodologies, advantages, and drawbacks of functional assays.

Cell Environment	Test	Instrument	Principle of the Assay: Endpoint	Advantages	Drawbacks
**Whole Blood**	Heparin-Induced Multiple Aggregometry (HIMEA)	Multiplate^®^ analyzer	measurement of impedance increase due to platelet aggregation and deposition onto the electrodes	- no need for platelet isolation (whole blood) - easy to perform and rapid assay (one hour) - very good sensitivity: potential alternative to SRA	- need for each laboratory to determine its own cut-off - need for collection of donors’ blood into tubes containing hirudin
**Platelet-Rich Plasma**	Platelet Aggregation Test (PAT)/Light Transmission Aggregometry (LTA)	aggregometer (light transmission)	measurement of increase in light transmission due to platelet aggregation	- easily available in many laboratories	- performance highly dependent on the donor′s platelet (role of plasma environment) - time-consuming - large volumes needed
	Flow Cytometry (FCM)	flow cytometer	measurement of increase in P-selectin or phosphatidylserine expression on an activated platelet (HIT Confirm^®^ assay and HIT Alert^®^)	- commercialized marked- in vitro diagnostic assay - performed in 2–3 h	- lower sensitivity than the reference test (i.e., SRA) - performance highly dependent on the donor’s platelet (role of plasma environment)
**Washed Platelets**	Serotonin Release Assay (SRA)	scintillation counter (β-radioactivity)	measurement of ^14^C-serotonin release from dense granules upon platelet activation	- rid of the inhibitory effect of plasma on platelet activation - considered “gold standard”	- platelet washing steps - use of radioelements (costly, specific agreement and premises) - time-consuming method: delayed results
	Heparin-Induced Platelet Aggregation (HIPA)	96-well plate with visual reading	visible macro-aggregates	- rid of the inhibitory effect of plasma on platelet activation	- platelet washing steps - lack of data on the performance - subjectivity of visual reading

**Table 2:** Most frequent volumes (μL) of components used in HIT functional assays.

	Platelets	Patient’s Sample	Heparin	Other	Final Volume	Final Sample Dilution
PAT	135 (PRP)	90	25	0	250	1/2.7
SRA	75 (WP)	20	5	0	100	1/5
HIPA	75 (WP)	20	10	0	100	1/5
FCM (HIT Confirm^®^)	10 (PRP)	10	5	20 (labelled antibodies + buffer)	50	1/5
HIMEA	300 (WB)	200	20	100 (saline)	620	1/3

Abbreviations: PAT: platelet aggregation test, SRA: serotonin release assay, HIPA: heparin-induced platelet activation, FCM: flow cytometry, HIMEA: heparin-induced multi-electrode aggregometry, PRP: platelet-rich plasma, WP: washed platelets, WB: whole blood.

**Table 3:** Diagnostic performances of the functional tests for the diagnosis of HIT.

Functional Assay	Study	Positivity Criteria of the Test	Patients Studied	Criteria for the Diagnosis of HIT	Diagnostic Performance (*)	Comparison
**PAT**	A. Greinacher et al. [59]	Platelet aggregation > 25% with LCH and no aggregation in the presence of the buffer (4 random donors)	209 patients with suspicion of HIT	A positive reaction with platelets of 2 or more donors	Not applicable (NA)	HIPA performed with platelets from same donors. Poor agreement between HIPA and PAT.
	B. Chong et al. [33]	Platelet aggregation > 25% with LCH	Thrombocytopenia due to other causes (*n* = 20) Non-thrombocytopenic patients who received heparin (*n* = 17) Patients with HIT (*n* = 17) Healthy donors (*n* = 23)	Clinical diagnosis; *n* = 17	Ss 39% with the least reactive donor Ss 81% with the most reactive donor Sp: 90%	with SRA
	C. Pouplard et al. [60]	Platelet aggregation > 20% with LCH and with a sharp slope (5 random donors)	100 patients with clinical suspicion of HIT	Clinical diagnosis; *n* = 40	Ss: 91% Sp: 77%	NA
	V. Galea et al. [62]	Maximal aggregation > 25% with LCH, no response in the presence of saline, and platelet aggregation inhibited with HCH	200 consecutive patients with clinical suspicion of HIT	Clinical context and positive SRA; *n* = 21	Ss: 76% Sp: 96% PPV: 80% NPV: 97%	NA
	J. Brodard et al. [61]	Platelet aggregation > 50% with LCH and with two out of four selected platelet donors	122 patients with clinical suspicion of HIT and positive anti-PF4/H ELISA	Clinical context and positive HIPA; *n* = 39	Ss: 69% Sp: 100%	NA
**Flow Cytometry Assay**	A. Tomer et al. [63]	Annexin V binding. ≥6.6% platelet activation with LCH and inhibition with HCH Grey zone 6–6.6%. The number of platelet donors is not mentioned	25 patients with clinical suspicion of HIT	Clinical diagnosis + and positive SRA; *n* = 19	Ss: 95% Sp: 100%	SRA
	S. Poley et al. [64]	Annexin V binding. >13% platelet activation with LCH and inhibition of platelet activation with HCH. Pooled platelets of selected donors	248 patients with clinical suspicion of HIT	Clinical diagnosis and positive HIPA; *n* = 17	Ss: 95% Sp: 96%	HIPA (4 donors)
	HS. Garritsen et al. [65]	Annexin V binding (HIT Alert^®^). ≥7.6% platelet activation in the presence of LCH and platelet activation reduced by ≥50% in the presence of HCH. One selected platelet donor	346 patients with clinical suspicion of HIT	Clinical diagnosis; *n* = 17	Ss: 88.2% Sp: 99.1%	For IgG ELISA negative sera: 98% agreement with HIT Alert^®^. For IgG ELSA positive sera: 52.7% agreement with HIT Alert^®^
	F. Mullier et al. [66]	Ratio PMP annexin V expression (LDH/HDH). One platelet donor only	53 patients with clinical suspicion of HIT	Clinical diagnosis; *n* = 9	Ss: 88.9% Sp: 100%	NA
	E. Malicev et al. [67]	>10% CD62P-positive platelets at LCH and ≥50% and inhibition of platelet activation at HDH. Two platelet donors	41 patients with clinical suspicion of HIT and positive ELISA IgG	Clinical context and positive HIPA; *n* = 14	Ss: 82% Sp: 83%	NA
	B. Tardy et al. [68]	P-selectin expression. >16.5% platelet activation with LCH and inhibition with HCH Two selected platelet donors	228 patients with clinical suspicion	Expert opinion adjudication (clinical diagnosis + local laboratory results); *n* = 106	Ss: 83% Sp: 97%	NA
	M. Cipok et al. [69]	≥2-fold greater P-selectin expression than that of the normal control. One platelet donor only	63 patients with clinical suspicion	Positive SRA; *n* = 21	Ss: 90.5% Sp: 95%	NA
	K. Althaus et al. [70]	P-selectin expression (Emo-test HIT^®^). %HEPLA: >13.0%. Grey zone 9.6–13%. One unselected platelet donor only	164 surgical or medical patients with clinical suspicion of HIT and positive EIA IgG	Positive HIPA; *n* = 33	Ss: 69.7% Sp: 75.4%	NA
**HIPA**	A. Greinacher et al. [27]	HIPA was positive if the suspension became transparent with LCH, but not with heparin HCH (4 random donors)	34 patients with suspicion of HIT	Not applicable	Not applicable	Excellent agreement with SRA: Kappa = 0.85 Moderate agreement with PAT: Kappa = 0.46
	P. Eichler et al. [71]	HIPA was positive if the suspension became transparent with LCH, but not with HDH. A sample was judged positive if positive results were obtained with test platelets of at least 2 of the 4 donors	Workshop involving 9 laboratories with 8 samples: 2 from healthy blood donors, 5 from HIT patients (with HIT antibodies), 1 from a patient with sepsis	Not applicable	Not applicable	Expected results in 82% of cases
**HIMEA**	M.C. Morel-Kopp et al. [34]	ISTH criteria	181 patients with suspicion of HIT and positive EIA	Clinical context and positive SRA; *n* = 72	Ss: 90.3% Sp: 89%	HIMEA and SRA were performed with the same good responder donors
	V. Galea et al. [62]	AUC with LCH > 267 AU with a representative shape of a platelet aggregation curve and a decrease in the AUC value with HCH > 50%	200 consecutive patients with suspicion of HIT	Clinical context and positive SRA; *n* = 21	Ss: 81% Sp: 99% NPV: 98% PPV: 89%	NA
	V. Minet et al. [72]	Platelet aggregation occurred in the presence of LCH with a reduction of >80% with HCH	116 patients with suspicion of HIT	4Ts score and Accustar HIT; *n* = 2	Ss: 100% Sp: 90%	NA
	J. Jin et al. [73]	AUC > 50 with LCH and AUC = 0 or inhibition of at least 50% of the AUC obtained with HCH	70 patients with suspicion of HIT	4Ts score > 4 and positive EIA IgG and positive SRA; *n* = 7	Ss: 85% Sp: 98%	NA
	V. Galea et al. [74]	Aggregation curve at LCH was typical and AUC decreased by 50% or more with HDH	87 patients with suspicion of HIT	Clinical context, positive SRA, and positive IgG ELISA; *n* = 12	Ss: 91% Sp: 100%	NA
**SRA**	D. Sheridan et al. [52]	Release > 20% with LCH and < 20% with HCH. One donor	28 patients with suspicion of HIT 573 non-HIT patients	Clinical diagnosis: *n* = 6	Ss: 100% Sp 99%	NA
	C. Pouplard et al. [60]	Release > 20% with LCH and < 20% with HCH. One donor	100 patients with suspicion of HIT	Clinical diagnosis: *n* = 40	Ss: 88% Sp: 100%	NA
	B. Chong et al. [33]	Release > 20% with LCH and < 20% with HCH. One donor	Thrombocytopenia due to other causes (*n* = 20)	Clinical diagnosis: *n* = 17	Ss: 65% with the least reactive donor Ss: 94% with the most reactive donor Sp: 90%	Comparison with PAT, Kappa = 0.60
	F. Mullier et al. [66]	Release > 20% with LCH and < 20% with HCH or less than 50% of that observed with LCH.	53 patients with suspicion of HIT	Clinical diagnosis: *n* = 9	Ss: 88.9% Sp: 95.5%	NA

Abbreviations: AU: arbitrary units; AUC: area under the curve; ELISA: enzyme linked immunosorbent assay; HCH: high concentration heparin; HIPA: heparin-induced platelet aggregation; ISTH: International Society on Thrombosis and Haemostasis; LCH: low concentration heparin; NA: not applicable; NPV: negative predictive value; PPV: positive predictive value, Sp: specificity; Ss: sensitivity; SRA: serotonin release assay, % HEPLA: heparin platelet activation index; (*) Proper determination of the diagnostic performance is crucial, but difficult (see text). Moreover, the number of patients being low, 95% confidence interval is large.
